# The Role of Androgens and Androgen Receptor in Human Bladder Cancer

**DOI:** 10.3390/biom11040594

**Published:** 2021-04-18

**Authors:** Elizabeth Martínez-Rojo, Laura Cristina Berumen, Guadalupe García-Alcocer, Jesica Escobar-Cabrera

**Affiliations:** Unidad de Investigación Genética, Posgrado en Ciencias Químico Biológicas, Facultad de Química, Universidad Autónoma de Querétaro, Querétaro 76010, Mexico; elizaro4309@gmail.com (E.M.-R.); lcbsq@yahoo.com (L.C.B.); guadalugar@yahoo.com.mx (G.G.-A.)

**Keywords:** androgens, androgen receptor, bladder cancer, signaling pathways, therapies

## Abstract

Bladder cancer (urothelial carcinoma) is one of the most frequently diagnosed neoplasms, with an estimated half a million new cases and 200,000 deaths per year worldwide. This pathology mainly affects men. Men have a higher risk (4:1) of developing bladder cancer than women. Cigarette smoking and exposure to chemicals such as aromatic amines, and aniline dyes have been established as risk factors for bladder cancer and may contribute to the sex disparity. Male internal genitalia, including the urothelium and prostate, are derived from urothelial sinus endoderm; both tissues express the androgen receptor (AR). Several investigations have shown evidence that the AR plays an important role in the initiation and development of different types of cancer including bladder cancer. In this article, we summarize the available data that help to explain the role of the AR in the development and progression of bladder cancer, as well as the therapies used for its treatment.

## 1. Introduction

Bladder cancer has high incidence and mortality around the world. Approximately, half a million new cases and 200,000 deaths have been reported worldwide per year [1]. In the United States, it is the fourth leading cause of death in men. About 83,730 new cases and 17,200 deaths from this pathology among men and women have been estimated [2]. Most of the people affected by this pathology are men over 65 years of age and, to a lesser extent, women. The main risk factors are smoking, exposure to chemicals, radiation therapy, and chemotherapy [3]. Androgens are classically known for their role in the male reproductive system. Androgens and their receptors control the transcription of genes associated with processes such as cell cycle, survival, and differentiation; thus, the study of their influence on the development, progression, and diagnosis in different types of cancer is very important [4]. Different studies have shown that the presence of androgens and their receptor is strongly associated with bladder cancer [5].

The aim of this review is to collect the most recent information on the role of androgens and the AR in the development and progression of bladder cancer, as well as the signaling pathways involved and new therapies.

## 2. Structure of Androgens and Androgen Receptors

Androgens are a group of steroid hormones that include mainly testosterone, which is converted to 5α-dihydrotestosterone (DHT, its active metabolite which shows a 2–5 times higher binding affinity to the AR than that of testosterone) through the action of the enzyme 5α-reductase [4]. Androgens are produced from the transformation of cholesterol in the testes (Leydig cells), ovaries, adrenal gland [4,6] muscles, fat tissue, skin, and endometrium [7].

The AR belongs to the steroid receptor family (NR3C4 of the 3-ketosteroid receptors, family III), one of the subtypes of the nuclear receptor superfamily, the latter composed of more than 500 transcription factors modulating gene expression [8,9,10]. The AR is composed of four different domains; an *N*-terminal domain (NTD), which contains the activation function-1 (AF-1) domain, which contains essential sites of phosphorylation; a DNA-binding domain (DBD) that has two zinc fingers, and its function is the recognition, dimerization, and binding to the DNA in androgen responsive elements (ARE); a hinge, a lysine rich region that is important for nuclear receptor localization; and a ligand-binding domain (LBD) that contains the AF-2 [6,11]. The AR gene is located on the long arm of the X-chromosome and is expressed in several tissues, including the prostate, breast, testes, skeletal muscle, uterus, and bladder [11]. The AR belongs to the family of transcription factors that homodimerize after ligand-binding, while RXR receptors may heterodimerize, and monomeric orphan receptors do not require dimerization [12,13].

## 3. Activation of Androgen Receptor and Cofactors

**Figure 1:**
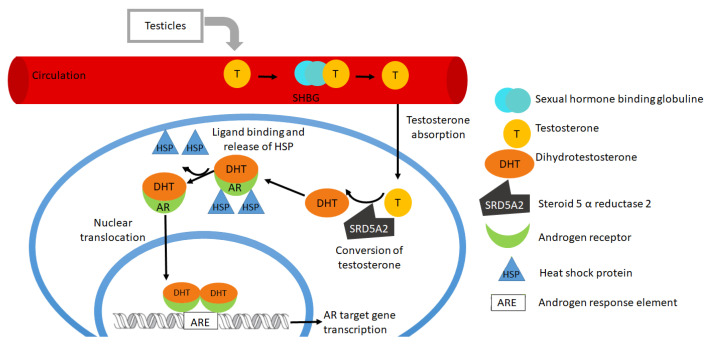
Genomic androgen receptor pathway.

In the absence of a ligand, the AR is bound to heat shock proteins that prevent the translocation to the nucleus and protect the receptors from degradation (and from the generation of toxic AR aggregates) and remains in the cytoplasm; after ligand binding, the AR dissociates from the heat shock proteins and translocates to the nucleus. In the nucleus, the AR binds to ARE located on the DNA promoter via zinc finger binding domains. Once the AR dimer is bound to the DNA, it forms complexes with transcriptional coactivators and co-regulators for the regulation of target gene expression (Figure 1) [4,6,14,15,16,17,18].

Coregulators are not DNA-binding proteins, but are recruited to transcription factors, such as the AR, and have different activities involved in the regulation of gene expression; some of them cause chromatin reconfiguration or are needed to bind some other cofactors [19].

**Figure 2:**
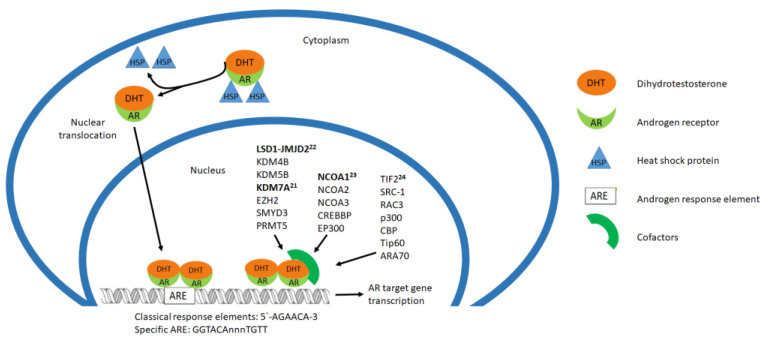
Cofactors involved in the regulation of AR target gene expression.

Liu et al., 2017 studied the AR coregulators in association with prostate cancer; they worked with 181 coregulators interacting physically and functionally, with AR 51 of them associated with prostate cancer and 22 correlated with the most aggressive characteristics (Figure 2). They managed the knockout of the 22 coregulators and identified four of them that decreased AR protein expression (GAK, HIP1, RAD9A, and SMAD3). They confirmed the participation of the other 18 coregulators, silencing each factor individually with specific siRNA, with p300 knock-down having the greatest affect. They found two novel transcriptional codes with WDR77-p53 and STAT3-IRF1 and the coregulation activity of PGAM5 on the AR; they described the intracellular heterogeneity of coregulator recruitment for specific gene targets [20].

The AR interacts with enzymes (cofactors) involved in the regulation of histone methylation, such as lysine-specific demethylase 1 (LSD1), KDM4B, KDM5B, KDM7A, EZH2, SMYD3 and PRMT5. Lee et al., 2020 found that the interaction of AR and KDM7A suggested that this histone demethylase mediated the growth of bladder cancer [21]. Kauffman et al., 2011 previously identified the association of bladder cancer with the AR-epigenetic coregulators: LSD1 and members of the Jumonji-domain containing coregulator family (JMJD2). The LSD1-JMJD2 complex with activated AR removes the methylation of histones and pharmacologic inhibition of LSD1 suppresses androgen induced transcription and bladder cancer cell growth [22]. Boorjian et al., 2009 looked for the participation of the coactivators NCOA1, NCOA2, NCOA3, CREBBP and EP300 with the AR activation urothelial carcinoma cells, finding their expression in bladder cancer and the differential regulation of NCOA1 [23].

Fancher et al., 2019 also report androgen receptor coactivators that have elevated expression in patients with prostate cancer, such as transcriptional intermediary factor 2 (TIF2), steroid receptor coactivator (SRC)-1, RAC3, p300, CBP, Tip60, and ARA70 [24].

The AR interaction with small molecules might allosterically modify the recruitment of coregulators, and the posttranslational modifications of every protein in the transcription complex might also be involved in differential responses; thus, the result might be confounding if the experimental designs do not consider these factors. Bisphenol A, as an environmental endodisruptor, can bind AR and is considered a direct xeno-estrogen AR agonist. The participation of these environmental ligands, as well as individual mutations or expression levels of steroidogenic genes, may explain the inter-individual variability seen in the AR activity related to prostate cancer and other pathologies [19].

The post-translational modification of receptors (and co-factors, as well as all proteins involved in steroidogenic signaling) may open a wide range of possibilities of protein–protein interactions, beyond the changes in conformations that other ligands can perform, as well as the expression of different splice variants of each protein or mutated genes [25,26,27,28]. Several variants, mutations, or even genomic rearrangements (*TMPRSS2-ERG* fusion is the most common in human prostate cancer, but is absent in small cell carcinoma of the bladder) are involved in pathophysiological situations correlated with AR signaling pathways; the complex interaction of each protein in a specific context may lead to different results, and represents a new pharmacological target for these pathologies, including bladder cancer [29]. New strategies that modulate AR signaling are developing, as demonstrated in the studies with long noncoding RNAs (lncRNAs), microRNAs, enhancer RNAs (eRNAs), and others [30,31,32,33].

## 4. Physiological Role of Androgen Receptors

The expression and action of androgens and their receptors has been observed in different tissues, including the prostate, testes, ovaries, endometrium, kidneys, liver, the cardiovascular system, the brain, the nervous system, skeletal muscle, skin and urinary bladder [4,34].

In men, free testosterone is only found between 1 and 2% of the total, 40% is transported attached to the albumin and the rest is coupled to the sex hormone binding globulin (SHBG) [4]. Androgens in men participate in processes such as the differentiation and development of the prostate [14]. During the fetal period, androgens participate in the masculinization of the fetus. A lack of androgens during the fetal stage can lead to disorders of sexual development. In infancy, the lack of expression of androgen receptors in Sertoli cells inhibits spermatogenesis, although high amounts of androgens are present. In adolescence, the increase in testosterone concentration induces the maturation of Sertoli cells and, consequently, an increase in the expression of the androgen receptor, initiating spermatogenesis. Other effects of androgens in this stage are the enlargement of the penis and scrotum and an increase in body hair. Androgens have anabolic activity in the bones, effects on muscle mass and adiposity, and have an influence on libido and sexual behavior [35].

In women, androgens are produced in different tissues such as the skin, but mainly in the ovaries, and are transported in the blood together with the SHBG or albumins [7]. The androgens present normally in women are androstenedione, dihydrotestosterone, dehydroepiandrosterone and dehydroepiandrosterone-sulfate, and testosterone [36]. Testosterone regulates the metabolism, strengthens muscle and bone, and maintains genitourinary health, mood, and some cognitive functions [7]. In women, androgens also have effects on hair cells, causing disorders such as acne, alopecia, and hirsutism, and alterations in the endocrine system such as polycystic ovary syndrome and adrenal hyperplasia [36]. Androgens have been shown to be stable during the menstrual cycle and form part of the circulating androgen pool; the concentration of androgens decreases with advancing age until before menopause [37]. After menopause, testosterone, together with estrogen, direct cell proliferation and preserve the vaginal epithelium. Between 20 and 30 years of age, there is the highest secretion of androgens and their precursors; later, their synthesis decreases by 50% at 40 years and up to 25% at 50 years of age. Before and after menopause, androgens intensify insulin resistance (particularly in obese women), which favors the growth of facial hair and increases hair loss. Emotional state and sexual behavior are regulated by sex hormones during a woman’s life; a decrease in androgens causes a loss of libido and a feeling of well-being. In addition, it induces changes in the genitourinary system, such as changes in the vulva, clitoris, urethra, and vagina; in the latter, it causes narrowing, inflammation or bleeding, and a decrease in acidity which favors infections by bacteria and fungi, which could later lead to urinary infections [7].

In men and women, the participation of ARs in the bladder is still under study, and although this organ is not considered responsive to androgens [14], few articles have reported the presence of AR in the normal human bladder. RAs have been identified in normal human bladder tissue during the fetal stage [38], and in adult women [39] and men [40]. In adult animal models, the presence of ARs has also been observed in the normal bladder [14]. In male rats, testosterone supplementation induces an increase in bladder mass and bladder mass smooth muscle cells [41]. Conversely, the deficiency of androgens and their receptors in rats induce histological changes in the bladder, modifies the smooth muscle bladder mass [42], affects the autonomic nervous system by reducing its function, and reduces the capacity of the bladder [14]. In an opposite way, the castration causes a significant decrease in bladder size [41]. These findings suggest that the AR signaling pathway could be important for normal bladder development and function.

On the contrary, several researchers reported a negative expression of ARs in normal adult human bladder in both men and women [43,44].

## 5. Classification of Bladder Cancer and Its Risk Factors

There are different types of bladder cancer; the most common type is urothelial carcinoma (also known as transitional cell carcinoma) and is originated in the urothelial cells that line the inside of the bladder [2]. Bladder cancer is classified into two different categories—non-muscle-invasive bladder cancer and muscle-invasive bladder cancer. The non-muscle-invasive bladder cancer refers to the invasion of one of the first two layer of the bladder, the urothelium or the lamina propria; and the muscle-invasive bladder cancer invades the third layer, the muscularis propria. [45]. These two categories can be classified as a high-grade or low-grade disease depending on the histomorphological characteristics [1]. The most frequently used classification is the American Joint Committee on Cancer (AJCC) TNM system. The TNM indicates the extent of the primary tumor in the bladder wall and whether it has reached adjacent tissues (T), spread to glands or lymph nodes near the bladder (N), and if the cancer has metastasized to distant organs (M). TNM staging is combined with numbering from 0–4; the higher the numbering, the greater the degree of the advancement of cancer [45].

There are several risk factors for the development of bladder cancer; the most common risks are tobacco smoking and old age (people over 70 years old), for its reduced ability to repair DNA damage produced by chemical compound such as aromatic amines, aniline dyes and others. The other risk factor is gender—the incidence of bladder cancer is four times higher in men than women [1]. Lifestyle, environmental, or occupational factors, such as smoking and industrial chemicals, are believed to contribute to this sex disparity [46]. Nevertheless, the expression and androgen receptor activity are correlated with bladder cancer [47,48,49,50]. This correlation could be explained due to the fact that AR signaling negatively regulates the activity of the uridine 5-diphodphoglucoronosyltransferase (UGT) enzymes. The UGT enzymes are responsible for metabolizing carcinogenic compounds such as aromatic amines [51] and, as their activity is diminished, carcinogenic compounds are not efficiently metabolized, promoting the development of bladder cancer. The combination of risk factors of prevalence in men such as smoking and AR signaling could explain the higher incidence of bladder cancer in men than in women. Several studies have identified the presence of AR as being between 30 and 80% in the cases analyzed. These data analyzed by gender showed a male predominance in the presence of AR [23]. This could suggest that the AR may participate in the development of bladder cancer, but not to be a determining factor.

Other factors such as race, cancer treatments such as radiation, some antineoplastic drugs such as cyclophosphamide and alkylating agents, inherited conditions such as Lynch syndrome, chronic urinary tract infections, the chronic use of urinary catheters, bladder stones, and parasitic diseases such as schistosomiasis in combination with viral infections such as papilloma virus increase the risk of bladder cancer [45,46,52].

## 6. AR Signaling in Bladder Cancer Carcinogenesis: Initiation and Promotion

Male internal genitalia, including urothelium, as well as prostate, are derived from the urothelial sinus endoderm and these tissues require the AR signaling pathway for its differentiation and development. Based on the common origin, a hypothesis has been proposed that AR signaling is involved in the carcinogenesis of bladder cancer [48,49,50]. The presence of ARs is associated with the development of bladder cancer. Several studies reported that the expression of the AR in malignant cells of the urothelial tissue ranges between 17 and 78% [46].

The AR plays a role in bladder cancer through many mechanisms that involve the steps of carcinogenesis. such as the regulation of tumor suppressors; the modulation of enzymes activity, non-genomic pathways, miRNAs; and processes of homeostasis such as autophagy. Carcinogenesis is a dynamic process that initiates within cells life spans; the process consists of three steps: initiation, promotion, and progression [53].

Initiation: Mutations, loss of alleles in the AR gene, and alteration of mRNA sequences are of high importance for the development of this type of cancer [46]. 4-(Methylnitrosamino)-1-(3-pyridyl)-1-butanone (NNK) is one of the carcinogens from tobacco, related to the development of bladder cancer. NNK is metabolized to 4-(methylnitrosamino)-1-(3pyridyl)-1-butanol (NNAL). NNAL is glucuronidated and excreted in the urine. One of the subfamilies of the UDP-glucuronosyltransferases (UGTs), UGT1A, contributes to the detoxification of bladder carcinogens. UGT1A has been identified as an androgen–responsive gene in prostate cancer cell lines. Human urothelium cell line SV-HUC transfected with wild-type AR and treated with DHT showed a decrease in all the subtypes of UGT1A expression; these effects were antagonized by an antiandrogen hydroxyflutamide (HF). These observations suggest that androgen–mediated AR signals promote bladder carcinogenesis by down-regulating the expression of UGTs in the bladder [54].

FOXO 1 is a tumor suppressor which binds directly to the AR. The inhibitory effect of FOXO 1 is dependent on its phosphorylation status; in a dephosphorylate state, FOXO 1 could bind to the full-length AR and active splice variants of the AR, inhibiting the transcriptional activity of the AR. However, the protein kinase B (PKB or AKT) signaling can induce FOXO 1 phosphorylation, which causes translocation from the nucleus to the cytoplasm, favoring transactivation of the AR in the nucleus [55]. Ide et al., 2020 investigated the role of FOXO 1 in the carcinogenesis of bladder cancer in relation to the AR and observed that in non-neoplastic urothelial cells (SV-HUC) or bladder cancer cell lines, AR expression or dihydrotestosterone (DHT) treatment causes a downregulation of *FOXO 1* gene and induced the inactivation of FOXO 1 through phosphorylation, FOXO 1 inactivation is correlated with the cell proliferation, migration and invasion of bladder cancer cells [56].

Long non-coding RNAs (lncRNAs) are a class of transcripts, >200 nucleotides in length, without the capacity to encode a protein. One of the lncRNAs is the X-inactive-specific transcript (XIST), which can function as an oncogene or tumor suppressor gene in some types of cancer, including hepatocellular carcinoma, prostate cancer, and bladder cancer [57,58]. The mechanism by which lncRNA XISTs exert their effect involves an interaction with miRNA, acting as a molecule sponge to sequester miRNA and indirectly increase oncogene expression [59].

Autophagy is a complex recycling pathway crucial for homeostasis. It has been demonstrated that in tumorogenesis initiation, autophagy can be tumor-suppressive [60,61]. A complex formed by autophagy related proteins (ATG) such as ULK1, ULK2, ATG13, RB1CC1/FIP200, and ATG101 induces autophagy. In bladder cancer cells, UM-UC-3, the AR influences autophagy by regulating ULK2; in presence of an AR knockdown, the mRNA and protein level of ULK2 are up-regulated, but with DHT treatment, the protein expression of ULK2 decreased. Inactivated AR signaling inhibits bladder cancer tumorigenesis via autophagy [62,63].

Promotion: Studies show that ARs increase the protein level and activity of the epidermal growth factor receptor (EGFR) as well as Herb2, promoting the development and progression of bladder cancer through the EGFR / ERBB2 pathway [46].

In AR-positive bladder cancer cells, DHT could activate the epidermal growth factor receptor (EGFR)/ERBB2/ERK pathway in the presence of the epidermal growth factor (EGF). One of the downstream targets of the ERK is the activating transcription factor 2 (ATF2), a member of the activating protein-1 (AP1) transcription factor family. ATF2 is a mediator of carcinogenesis of several types of neoplasms which can be phosphorylated by ERK on Thr71. Treatment with DHT in AR-positive bladder cancer cells induces the expression of p-ERK and also induces the expression of p-ATF2, but if any change in ATF2 protein expression is observed, these effects could be antagonized by HF. These findings suggest that the AR pathway is involved in the tumor growth modulating ATF2 activity through ERK in bladder cancer cells [64]. 

Xiong et al. annual report details an interaction between XIST, a long non-coding RNA, and miR-124 that regulates bladder cancer cell growth, through directly targeting the AR. In the bladder cancer cells TCC-SUP, EJ, SW780, and UM-UC-3, XIST and AR are up-regulated. A knock down of *XIST* showed a decrease in AR protein expression and repressed cell proliferation; this means that XIST regulates AR expression, and both function as oncogenes in bladder cancer. In order to find the mechanism by which XIST regulates AR protein expression, a relation between miR-124 and XIST was observed; miR-124 has recently been confirmed as a tumor suppressor regulating proliferation, migration, and invasion in many cancers. TCC-SUP and UM-UC-3 cell lines showed an increase in miR-124 expression and a decrease in *AR* expression in response to *XIST* knock down, but in the presence of *XIST*, a decrease in miR-124 expression is observed and an increase in AR expression level; these data suggest that miR-124 functions as a tumor suppressor of bladder cancer by a regulation of the AR, and XIST serves as a sponge of miR-124, restoring the AR proliferative activity [65,66].

There are isoforms of the AR that do not contain LBD; they can be located in the cytosol and in the nucleus. Once in the nucleus, these variants can activate transcription to promote the proliferation. These variants have been identified in bladder cancer [67].

## 7. AR Signaling and Bladder Cancer Progression

The genomic pathway of the AR starts with the binding of DHT to the receptor in the cytoplasm; AR is released from heat shock protein and translocated into the nucleus, dimerizes, and then binds to the androgen response element [15]. However, many of the cellular responses do not require transcription mediated by the AR, because AR binding to DHT is able to associate with molecular substrates in the cytoplasm and membrane to activate intracellular kinase cascades; and these actions are known as the non-genomic signaling of the AR. Some of the signaling molecules activated by this signaling include Src, Ras, MAPK, Akt, PKC, PLC, and EGFR [68,69].

AR non-genomic signaling originates in the cytoplasm or at the plasma membrane. Some Western blot analyses show the intracellular localization in non-malignant transitional epithelial cells of the ureter HCV29 and in bladder cancer T24 cells. Without a ligand, the AR is located in the cytoplasm principally, but in presence of DHT, a small amount is present in the nucleus or in the cytoplasm, as well as, in the cytoskeletal and membrane; these observations suggest that DHT activates the genomic and non-genomic AR signaling in bladder cancer [68,70,71].

The AR has also been reported to participate in the activation of the epithelial–mesenchymal–transition (EMT), which is directly associated with metastasis. EMT is a biological process in which the cell undergoes biochemical changes, where it loses cell attachment through adhesion protein proteolysis, and the loss of apical–basal polarity, and undergoes cytoskeletal rearrangements, increasing its capacity for mobility, which allows invasion and metastasis [72]. EMT regulates via transcription factors such as TWIST, ZEB and Snail, which directly represses the expression of genes involved in cellular adhesion, polarity and cytoskeleton reorganization [73]. In addition to transcription factors, matrix metaloproteinases (MMP), zinc-dependent endoproteases, induced changes during EMT to facilitate invasion and migration [74]. One of the molecules activated by androgen/AR interaction is Akt, through an increase in its phosphorylation on Ser473, which is involved in proliferation and migration. Akt regulates the phosphorylation of eIF4E, which is an important regulator of mRNA translation and functions as a proto-oncogene involved in malignant transformation, proliferation, invasion, and metastasis. Phosphorylation on Ser 209 of eIF4E is required for the translation of Snail and MMP-3, two factors that regulate epithelial–mesenchymal transition and invasion [68,70,71].

**Figure 3:**
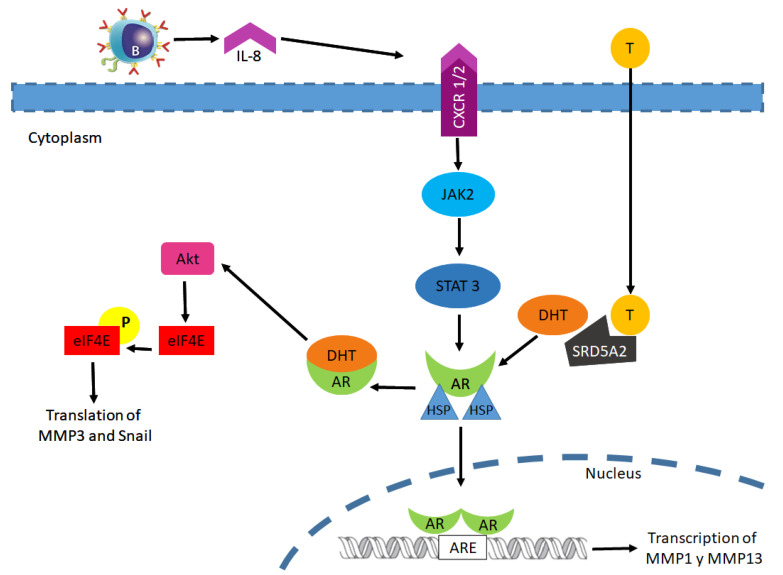
Androgen receptor non-genomic signaling pathway in the progression of bladder cancer.

The tumor microenvironment (TME) may play an important role in the progression of many cancer types. Infiltrating immune cells such as B lymphocytes (B cells) are vital components of the bladder cancer microenvironment [75]. B cells can secrete IL-8, a cytokine that is implicated in the activation of the AR and cancer progression through the androgen independent pathway in prostate cancer. Ou et al., 2017 show that the bladder cancer cells TCCSUP, T24, and J82 co-cultured with B cells increase their invasion capacity, proposing that recruited B cells could increase IL-8/AR signals. The androgen-independent pathway could result in the upregulated expression of metastasis genes, including MMP1 and MMP13 (Figure 3) [76].

Furthermore, there is evidence that ARs regulate urothelial cancer stem cells (UCSCs) in the development of bladder cancer by increasing the cancer steam cell (CSC) population and promoting EMT, both in vivo and in vitro [72].

A new axis AR/ADAR2/circFNTA/miR-370-3p/FNTA/KRAS for bladder cancer progression has been discovered. Upon DHT binding, the AR interacts specifically with ARE I/II in the promoter region of ADAR2 (adenosine deaminases acting on RNA), inducing its repression [77]. *ADAR2* is an RNA editing gene that encodes an enzyme whose function is to modify adenosine nucleotide into inosine by deamination [78]. Repression of *ADAR2* results in an increase in circFNTA levels, which leads to an elevated sponging of the miR-370-3p; this miRNA bind directly to the farnesyltransferase (FNTA) mRNA 3’ UTR binding site to increase *FNTA* expression. *FNTA* gene encodes farnesyltransferase, an important enzyme for farnesylation of the CAAX motif of cytosolic Ras; thus, high levels of FNTA can activate the KRAS gene to promote cell invasion [77,79].

Based on the evidence, androgens and ARs play an important role in bladder development; it is believed that blocking AR signaling may work in bladder cancer therapy [4]. On the other hand, resistance to chemotherapy is associated with the activation of androgen receptors [80].

## 8. Bladder Cancer Therapies

Suitable treatment of bladder cancer can be indicated after diagnosis following transurethral resection of the tumor with cytoscopy and urine cytology for screening and initial diagnosis and for the monitoring or surveillance of tumor recurrence or its progression; urine-based detection markers (nuclear matrix protein 22, bladder tumor antigen, carcinoembryonic antigen, bladder tumor cell-associated mucins and chromosomal alterations) are also useful tools [81,82,83,84].

The classification of bladder cancer according to the traditional American Joint Committee on Cancer (AJCC) takes tumor invasion as the major characteristic to define the cancer subtype: muscle (MIBC) or non-muscle invasive bladder cancer (NMIBC), the latter with better prognosis. The presence of nodal or distant metastases should be looked for to adjust the treatment accordingly, and several tools such as nomograms and evidence guidelines from cancer organizations for risk assignment can be used. Unfortunately, survival outcomes for bladder cancer patients have not changed in the last two decades; thus, research is needed to give better alternatives [82,85].

Personalized treatment is intended to be accurate after determining the specific subtype of cancer, considering gene expression profiles leading to specific drug responsiveness. Satyal et al., 2019 described different bladder cancer classification systems based on characteristic expression features of the different tumors (molecular subtyping): Lund taxonomy, UNC (University of North Carolina) classification, TCGA (The Cancer Genome Atlas) taxonomy, the MDA CC (MD Anderson Cancer Center) classification, the BOLD taxonomy, and the classifications proposed by Hedegaard et al. and Hurst et al. for different subtypes of non-muscle invasive bladder cancer (NMIBC). The genomic subtyping classifier (GSC) was also described by Seiler et al., which ended in the proposal of the Decipher Bladder tool [86,87,88,89].

Furthermore, Fong et al., 2020 described the efforts of the international team of experts for a consensus molecular classification (CMC) to offer studies in terms of clinical utility [83,90], therapeutics being one of the most important goals to improve. McConkey and Choi previously reviewed molecular subtypes of bladder cancer (2018), considering basal and luminal molecular subtypes, and pointed out a neuroendocrine phenotype of basal cancer associated with poor survival [90,91,92]. In this scope, the molecular subtypes may present differential sensitivities to neoadjuvant chemotherapy. Guo et al., 2020 proposed the use of protein expression signatures for luminal (GATA3) and basal (KRT5/6) markers [93].

After transurethral resection of the bladder tumor in NMIBC, repeated if needed, an adjuvant treatment is considered. Standard intravesical therapies include chemotherapy agents such as mitomycin C, gemcitabine, epirubicin, docetaxel, doxorubicin, valrubicin, cisplatin, and thiotepa, among others. Immunotherapy is also considered, where Bacillus Calmette–Guerin (BCG) is preferred, but several other treatments are used, such as interferon and immune checkpoint inhibitors [82,94].On the other hand, MIBC treatment may consist of neoadjuvant chemotherapy with cisplatin and cystectomy; combinations of cisplatin, methotrexate, and vinblastine chemotherapy with cystectomy and/or radiotherapy have been investigated and proposed with improvement outcomes, although eligibility for cisplatin-based chemotherapy should take into account several criteria to minimize the toxicity of the treatment. Radical cystectomy alone is the most used MIBC treatment, and neoadjuvant therapy before cystectomy is not considered to be standard for the trimodal treatment of MIBC [95,96,97,98].

BCG-based immunotherapy remains ineffective in some NMIBC patients, although efforts have been made to characterize the response, giving the opportunity of an early selection of strategies for the patients who do not benefit from BCG treatment [99]. The expression of several biomarkers related to immune response correlate with resistance to BCG treatment; immune checkpoint inhibitors are complements for the therapy, in particular PD-L1 inhibitors, such as atezolizumab, nivolumab, pembrolizumab, avelumab, and durvalumab [94].

Actual studies regarding immune interactions regulating treatment responsiveness, such as studies of exosomes (cancer-derived exosomes, oncosomes) and even their possible use in immune therapies, are increasing [100,101]. Liquid biopsy markers and urine–derived information might change the scope of cancer treatment, using new technology with higher sensitivity to acknowledge all of these molecule markers [102,103,104]. There are new proposals of panels and groups of biomarkers to identify bladder cancer subtypes, with state-of-the-art technology. Cavallari et al., 2020 have proposed a promising panel of microRNAs for the follow-up assays for patients after tumor resection. Liu et al., 2021 selected five mRNAs for the detection of the recurrence of NMIBC, whereas Sun et al., 2021 proposed an NGS-based panel as a surveillance method for bladder cancer, and Chen et al., 2020 studied the participation of cancer-associated fibroblasts with single-cell RNA sequencing focused on the immunosuppressive tumor microenvironment [105,106,107,108].

Kang et al., 2020 also illustrate the taxonomic classifications, with suggested treatments for different MIBC subtypes based on molecular biomarkers [109]. The combination of neoadjuvant therapy with immune checkpoint inhibitors (designed for suitable biomarker expression) is currently being tested and is expected to increase efficacy in treatments [110]. 

In the search for new therapy alternatives, male predominance is one of the important features which requires the consideration of the AR as a pharmacological target [82]. Nuclear receptors consist of a superfamily of proteins that includes receptors for steroids (androgens, estrogens, glucocorticoids, progesterone) and non-steroid receptors (thyroid hormones, vitamin D, retinoids, farnesoids, and others such as the peroxisome proliferator-activated receptors); steroids modulate (directly or indirectly by activation of their receptors) several key molecules, such as UDP-glucuronosyltransferases (UGTs), GATA3 zinc-finger transcription factor, FOXO1 forkhead transcription factor, CD24 cell adhesion molecule, β-catenin key component for Wnt signaling pathway, ELK1 transcription factor, ATF2 leucine zipper transcription factor, and NF-kB transcription factor [111,112].

Bladder cancer is not considered an androgen-driven malignancy, although some experimental models have shown that carcinogenesis can be dependent on AR signaling: wild type animals developed cancer with *N*-butyl-*N*-4-hydroxybutyl nitrosamine treatment, but AR knockout mice did not; some of the knockout animals developed cancer after supplementation with dihydrotestosterone, and treatment with flutamide (antiandrogen) suppressed the proliferation of xenograft tumors [14,113].

Gil et al., 2019 propose that dihydrotestosterone increases the risk of bladder cancer in men, based on the signaling pathways found in four bladder cancer cell lines [68]. Moschini et al., 2019 failed to associate androgen deprivation therapy with a subsequent risk of developing bladder cancer in men with prostate cancer, while Shiota et al. (2015) found a reduced comorbidity of bladder cancer after androgen-deprivation therapy compared to radiotherapy [114,115].

On the other hand, Sanguedolce et al., 2020 did find significant relevance for a correlation between the lack of androgen receptor expression and recurrent disease [116]. Yang et al., 2020 have reported the differential effects of the AR in the promotion of cancer metastasis in bladder cancer, while suppressing prostate cancer metastasis [117]. Yasui et al., 2019 also found that a high AR mRNA-expressing group tended to have a longer recurrence-free survival, and Katleba et al., 2021 found a low molecular weight variant of the androgen receptor in most bladder cancer malignancies, missing the ligand binding domain [67,118].

Several preclinical studies have tested the inhibition of ARs in bladder cancer, but clinical trials are still lacking to verify their efficacy; among two of the registered clinical trials, enzalutamide was shown to be safe when combined with gemcitabine and cisplatine (NCT02605863 was terminated) [14,119]. 

Promising therapies include the modulation of AR signaling, which can be modified in different ways, such as with ASC-J9, an enhancer of AR degradation that suppressed bladder cancer in pre-clinical trials (only clinical trials for acne are currently registered) and AZD 5312, an AR antisense oligonucleotide that has already completed phase I clinical trials for solid tumors [120,121,122,123].

A clinical trial that will analyze 75 approved anticancer agents for urothelial cancer, enlisting abiraterone, will provide more information on the efficacy of these agents in bladder cancer treatment [124]. Research is still lacking for small molecules that ortho- or allosterically interact with androgen receptors [125,126], as several other AR-targeted new drugs need to be investigated (such as RNA-based therapies), most of them for prostate cancer, although bladder cancer might also benefit from these [127].

## 9. Conclusions

Androgen receptor signaling is involved in bladder cancer, although this pathology is not considered sex-related, because the higher associated risk in men versus women may reflect other aspects, nor is the bladder considered an androgen-responsive organ [14,35,84]. All the components in AR signaling are studied to understand their participation as biomarkers or as pharmacological targets. Traditional orthosteric molecules, competing with androgens, or allosterical new molecules that might interfere with the dimerization or association of the AR with any of the cofactors it needs to fulfill the signaling pathway can be tested as coadjuvants in personalized treatment once the cancer is properly diagnosed. New ARN-based strategies and immunotherapy are expected complements for the treatment, although the prevention of the progression of bladder cancer might be one of the most important recommendations. Cigarette smoking is a very important bladder cancer risk-associated event [86]; thus, personalized neoadjuvant treatment might consider possible interactions of any of the proposed therapeutic molecules with this risk factor.

## Data Availability

Not applicable.
